# Cyclic Behavior of Mortarless Brick Joints with Different Interlocking Shapes

**DOI:** 10.3390/ma9030166

**Published:** 2016-03-04

**Authors:** Hongjun Liu, Peng Liu, Kun Lin, Sai Zhao

**Affiliations:** 1 Shenzhen Engineering Lab for Wind Environment and Technology, Shenzhen Key Lab of Urban & Civil, Engineering Disaster Prevention & Reduction, Shenzhen Graduate School, Harbin Institute of Technology, Shenzhen 518055, China; liuhongjun@hit.edu.cn (H.L.); liupeng2009.dream@163.com (P.L.); 2 Zhengzhou Architectural Design Institute, Zhengzhou 450052, China; zhaosai111@126.com

**Keywords:** mortarless brick joints, interlocking shapes, cyclic loads, shear-compression behavior, experiment

## Abstract

The framed structure infilled with a mortarless brick (MB) panel exhibits considerable in-plane energy dissipation because of the relative sliding between bricks and good out-of-plane stability resulting from the use of interlocking mechanisms. The cyclic behaviors of MB are investigated experimentally in this study. Two different types of bricks, namely non-interlocking mortarless brick (N-IMB) and interlocking mortarless brick (IMB), are examined experimentally. The cyclic behavior of all of the joints (N-IMB and IMB) are investigated in consideration of the effects of interlocking shapes, loading compression stress levels and loading cycles. The hysteretic loops of N-IMB and IMB joints are obtained, according to which a mechanical model is developed. The Mohr–Coulomb failure criterion is employed to describe the shear failure modes of all of the investigated joints. A typical frictional behavior is observed for the N-IMB joints, and a significant stiffening effect is observed for the IMB joints during their sliding stage. The friction coefficients of all of the researched joints increase with the augmentation of the compression stress level and improvement of the smoothness of the interlocking surfaces. An increase in the loading cycle results in a decrease in the friction coefficients of all of the joints. The degradation rate (*DR*) of the friction coefficients increases with the reduction in the smoothness of the interlocking surface.

## 1. Introduction

**Figure 1:**
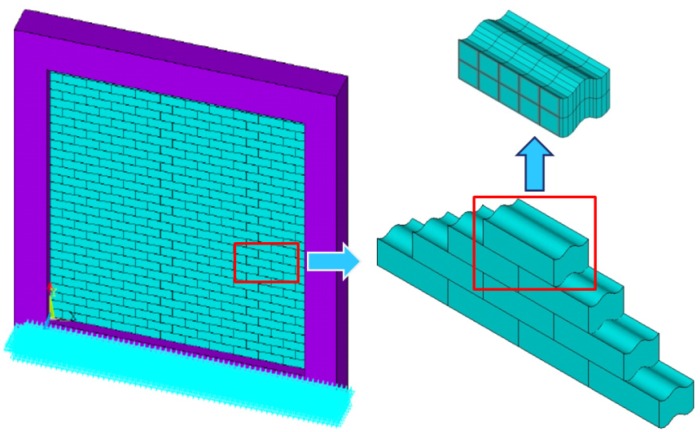
Reinforcement concrete frame with a mortarless brick (MB) panel.

Reinforced concrete (RC) frame structures with masonry panels are known for their economic feasibility and low technology requirement. However, their disadvantage in terms of seismic behavior is increasingly highlighted, especially in high seismic-prone regions [1,2,3,4]. To improve seismic behavior, a conceptually novel system for framed masonry panels was proposed in [5]. According to the concept, the frame was infilled with a mortarless brick (MB) panel. An MB panel can dissipate energy because of the relative sliding of each brick and avoid out-of-plane failure through the interlocking mechanism (see Figure 1). A series of experiments on an MB panel infilled frame was carried out by the authors, and the results indicated that the MB panel exhibits considerable energy dissipation during cyclic loading and can significantly improve the seismic behavior of the frame structure [5]. To evaluate the contribution of the energy dissipation and lateral resistance of the MB panel to the frame, an equivalent model was introduced in [6]; the authors found that the energy dissipation of the MB panel results from friction between bricks.

Improvement of the energy dissipation of the MB panel requires some knowledge of the particular mechanical properties of the novel component. However, previous research mainly focused on the shear and compression behaviors of traditional masonry with mortared brick units [7,8,9,10,11,12]; their results are unsuitable for dry stack masonry. Studies on the MB panel became active only in the last decade. However, these recent studies mainly focused on the cyclic behavior of non-interlocking mortarless brick (N-IMB) joints. Lourenco *et al.* [13,14] applied the couplet test to investigate the shear-compression behavior of dry stack stone joints. According to their research, the Mohr–Coulomb failure criterion can describe the characteristics of dry stone joints under different compression stress levels; the friction coefficients of the dry stone joints were obtained experimentally, and the cohesion for the investigated joints could be neglected. Zuccarello *et al.* [15] applied the traditional triple test and confirmed that the shear-compression behaviors of N-IMB joints represent the Coulomb friction law. Lin *et al.* [16,17] proposed a new modified loading device, which was improved through the traditional triple test, to study the cyclic behavior of N-MIB joints. However, the new loading device induced additional bending moments on the contact surface; the additional bending moments resulted in a pinch phenomenon in the hysteretic loops. Therefore, the proposed loading device should be improved to avoid the additional moments.

Different from the non-interlocking mortarless brick (N-IMB) panel, the interlocking mortarless brick (IMB) panel exhibits different in-plane/out-of-plane behaviors, damping ratios and energy dissipations [18,19,20,21]. Sturm *et al.* [22] and Rui *et al.* [23] studied the shear and compression behaviors of IMB experimentally. Their results indicated that the cohesion of IMB can be neglected, and pre-compression level and interlocking shapes are both important to the shear-compression behavior of the researched interlocking blocks. The cyclic behavior of IMB wall was investigated by Qu *et al.* [20]; severe damage was found at the bottom of the IMB walls because of large drift levels, which indicate that the interlocking shapes need to be improved. Thanoon *et al.* [24,25,26] studied the effects of the interlocking shapes of blocks on the compressive and out-of-plane behaviors of mortarless block masonry through numerical simulations and experimental tests; however, the influence on the in-plane behavior was not investigated.

The mechanical behavior of the contact between the mortarless bricks, which is a nonlinear problem, can become highly sophisticated by considering the influence of interlocking shapes. Ensuring accuracy during the processing and installation of mortarless bricks in practical engineering is difficult and causes a far less idealized contact between mortarless bricks under practical conditions than those under laboratory test conditions. Moreover, the use of interlocking bricks aggravates the non-idealized characteristics of the contact conditions for mortarless joints. Therefore, the frictional parameters (mainly the friction coefficient) and shear force-displacement hysteretic loops obtained from previously-reported shear-compression tests of N-IMB joints are unsuitable for the characterization of IMB joints. Obtaining in-depth insights into the shear-compression characteristics of IMB joints by considering the influence of different interlocking shapes is highly necessary.

A comprehensive experimental investigation of the behavior of IMB joints with four different interlocking shapes was carried out at Harbin Institute of Technology Shenzhen Graduate School. The present study aims to improve knowledge of the mortarless masonry structure under cyclic loading, which is of crucial importance in the evaluation of the energy dissipation of MB panels. Aside from those of interlocking shapes, the effects of compressive stress and loading cycles were also investigated and analyzed quantitatively.

## 2. Description of the Specimen

### 2.1. Design and Construction of the Brick

Light aggregate concrete (LAC) bricks were selected for the cyclic test. According to the material standard for LAC [27], the mix proportion (cement:fly ash:ceramsite:sand) of LAC was determined to be 1:0.27:1.7:2.6, with a water cement ratio (*w*/*c*) of 0.42. Then, three specimens of different mix proportions were selected for minor adjustments. The corresponding apparent density and seven-day compressive strength for each specimen are listed in Table 1. The No. 3 mix proportion, which meets the provision of the strength standard, was selected for the LAC brick specimens investigated in the subsequent experiments.

**Figure 2:**
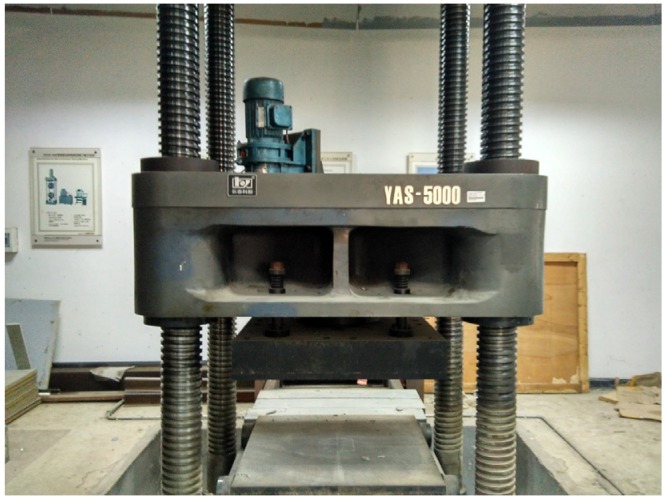
YAS-5000 compression-testing machine.

As shown in Figure 2, the YAS-5000 compression-testing machine (Changchun Kexin Test Instrument Company, Changchun, China) was utilized to perform the uniaxial test to determine the compressive strength of LAC. The obtained 28-day compressive strength and material density for 18 LAC cubic specimens are listed in Table 2. The average density (ρ = 1746 kg/m3) and average compressive strength (*f_c_* = 31.7 MPa) were obtained for LAC.

### 2.2. Dimension and Interlocking Shapes of the Brick

Although IMB can improve the out-of-plane stability of the MB panel effectively, unsuitable interlocking shapes or dimensions would lead to significant stress concentration and potential damage for the MB panel [28,29,30].

**Figure 3:**
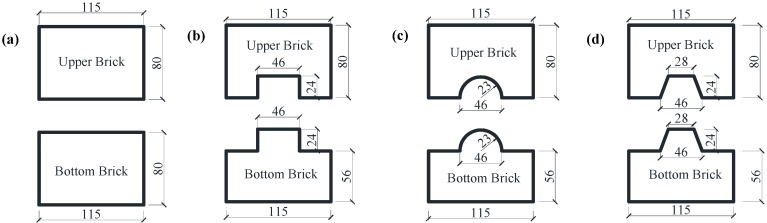
Dimensions and profiles of the cross-section for specimens of bricks with various interlocking shapes: (**a**) non-interlocking; (**b**) rectangular interlocking; (**c**) circular interlocking; (**d**) trapezoidal interlocking. (Units: mm).

**Figure 4:**
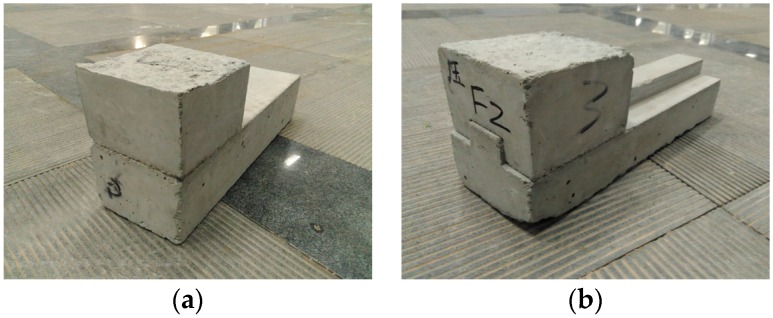

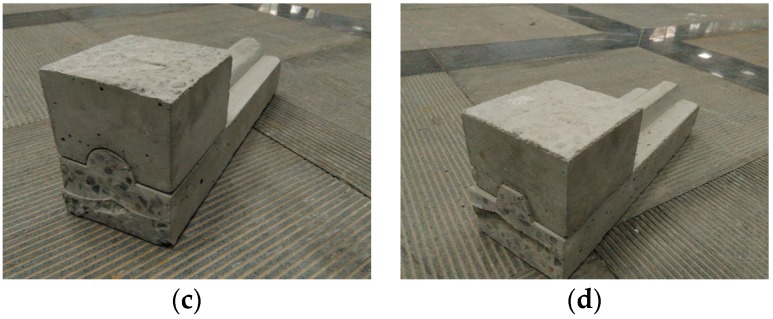
Photos for specimens of the bricks with various interlocking shapes: (**a**) non-interlocking; (**b**) rectangular interlocking; (**c**) circular interlocking; (**d**) trapezoidal interlocking.

In this study, the shear-compression characteristics of N-IMB joints and IMB joints with three interlocking shapes, namely rectangular, trapezoidal and circular, were investigated through the cyclic loading tests. The designed bricks are shown in Figure 3. The length, width and height of the bricks for the upper and bottom portions are 80 mm × 115 mm × 80 mm and 375 mm × 115 mm × 80 mm, respectively, as shown in Figure 4. The interlocking height and width ratio of IMB have been confirmed to be 0.3 and 0.4 according to previous research [28,29]. The dimensions and profiles of the cross-section for the non-interlocking and interlocking bricks are shown in Figure 3. Figure 4 shows photos of the test specimens of the bricks casted in-place in the laboratory.

## 3. Description of Testing Procedures

**Figure 5:**
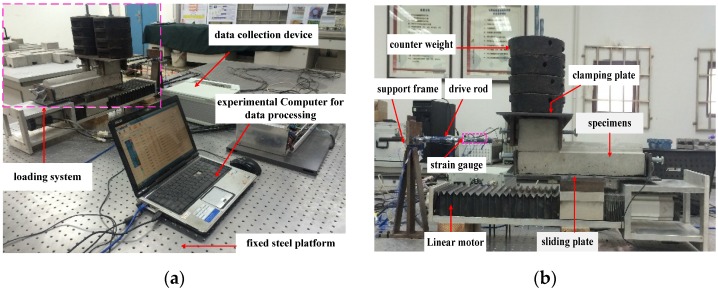
Test setup: (**a**) entire view; (**b**) loading system.

The test setup is shown in Figure 5. The upper brick is fixed in the steel clamping plate, which is connected to the support frame through a rigid drive rod. The bottom brick is fixed in the steel slot, which is bolted on the sliding plate of the loading device. The counter weights, which are employed to apply different vertical compression levels to the upper brick, are fixed on the top of the steel clamping plate through a steel rod. Both the support frame and loading device system are fixed on a steel platform.

A linear motor, which can achieve real-time control and provide cyclic horizontal displacement with constant speed, was utilized as the loading equipment. The loading speed of the linear motor ranges from 1 to 500 mm/s, and the acceleration time is 0.1 s with a repositioning accuracy of 0.02 mm.

The mass of each counter weight is 5.098 kg. The axial deformation of the linked drive rod caused by the horizontal right/left sliding of the bottom brick was measured with four strain gauges symmetrically plastered on the surface of the drive rod to avoid the effects of out-of-plane bending. The deformation (strain) was then converted to axial force of the drive rod, which is equal to the shear force of the sliding mortarless joint between upper and bottom bricks. Lastly, the frictional coefficients, which are equal to shear force divided by the area of the contact surface, were obtained. The resistance and sensitivity coefficients of the strain gauge are 120 ± 0.12 Ω and 2.12 ± 0.12, respectively. The DH5929 data collection device (with a measuring range of −1000 to +1000 με and sampling frequency range of 1 to 20,000 Hz) was utilized to collect the deformation (stain) data of the linked rod.

Prior to the test, the devices shown in Figure 5 were assembled together as an entire loading system. The leveling adjustment for all loading devices was enhanced during the assembling process to ensure that the brick slides in the same horizontal level all of the time. During the test process, leveling adjustment of the loading devices was carried out with a dial indicator. Real-time monitoring of the deformation of two supporting screw rods was employed to ensure the symmetry and level of the dial indicator. All of these measures guarantee the accuracy of the measured force.

This test involved 16 load cases. Four types of interlocking shapes (*i.e.*, non-interlocking, circular interlocking, trapezoidal interlocking and rectangular interlocking) and four compression stress levels (*i.e.*, 0.017, 0.028, 0.039 and 0.05 MPa) were investigated. Four loading cycles with an amplitude of 250 mm were applied to each loading case. The loading speed was set to 1 mm/s, which can be assumed as a quasi-static test.

## 4. Experimental Results and Discussion

### 4.1. Hysteretic Loops

**Figure 6:**
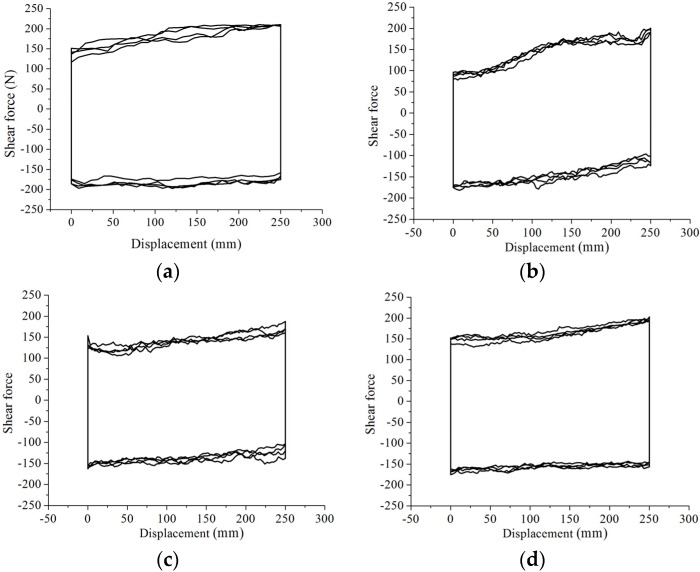
Typical hysteretic loops for the non-interlocking mortarless brick (N-IMB) and IMB joints under four cyclic experimental tests: (**a**) non-interlocking; (**b**) circular interlocking; (**c**) trapezoidal interlocking; (**d**) rectangular interlocking.

**Figure 7:**
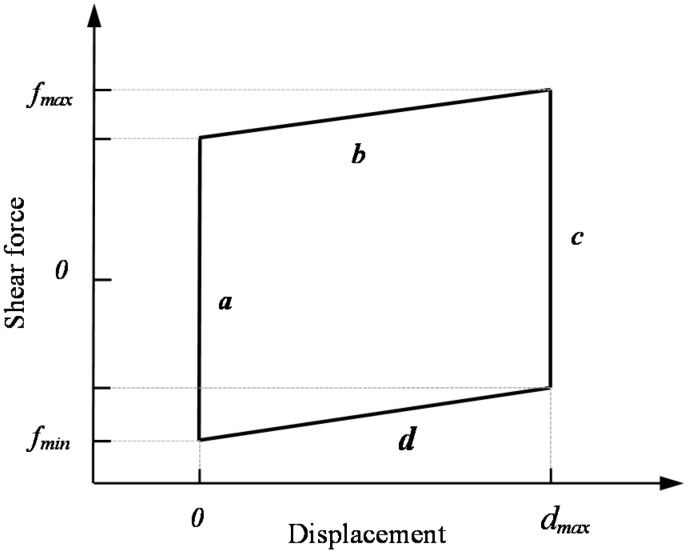
Mechanical model of the hysteric loops for the N-IMB and IMB joints.

Figure 6 shows the typical hysteretic loops of the N-IMB and IMB joints. A mechanical model was established and is shown in Figure 7. The results indicate that the hysteretic loop can be divided into four stages, namely initial loading (Stage a), constant loading (Stage b and Stage d) and unloading (Stage c). Compared to those of the N-IMB joints, the initial and unloading stages of the IMB joints exhibit similar characteristics. However, for the constant stage, the N-IMB and IMB joints exhibit a significant difference.

Unlike that in the N-IMB joints, the shear force of the IMB joints exhibits stiffness hardening behavior in the constant loading stage (Stages b and d); this behavior varied as the interlocking shape changed, shown as in Figure 6b–d. In consideration of the test process, it is concluded that the stiffness hardening behavior was caused by the loading direction being not parallel to the longitudinal extension of the interlocking portion during the loading process. Therefore, additional compression stress was generated; the shear force of the IMB joints increased; and the stiffness hardening behavior appeared.

### 4.2. Mohr–Coulomb Failure Criterion

The average value of the constant stage was selected to calculate shear and compression stresses as follows:

(1) $$\tau =\frac{F}{A}，\sigma =\frac{N}{A}$$

where τ is the shear stress, σ is the normal stress, *F* is the shear force that is equal to the average force of the constant loading stages, *N* is the normal force that is equal to the vertical applied force caused by the gravity effects of the countered weights and *A* is the vertical projection contact area in the experiment.

**Figure 8:**
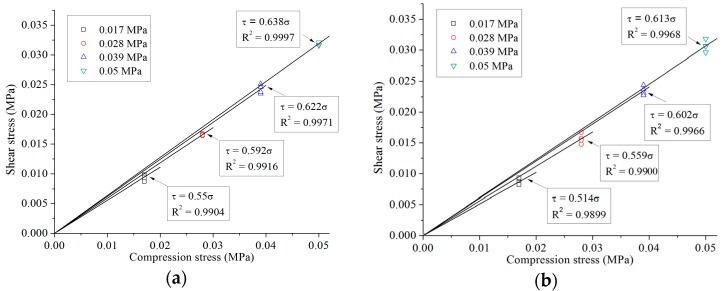

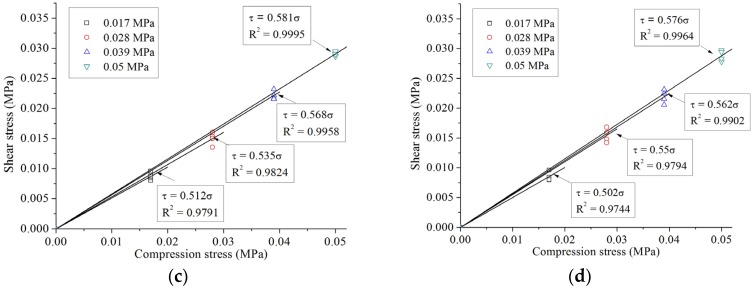
Experimental failure criteria for N-IMB and IMB joints under different compression stress levels: (**a**) non-interlocking; (**b**) circular interlocking; (**c**) trapezoidal interlocking; (**d**) rectangular interlocking.

The shear stress *versus* compression stress for all specimens under four-cycle loading is shown in Figure 8. As indicated by the figure, the relationship between shear stress and compression stress for both N-IMB and IMB joints under all researched compression levels complies with the Mohr–Coulomb failure criterion, namely,

(2) $$\tau =c_{0}+\mu \sigma$$

where *c*_0_ denotes initial cohesion, which can be assumed as zero for both N-IMB and IMB joints, and μ denotes the representation coefficient. The friction coefficients of both N-IMB and IMB joints with different interlocking shapes are listed in Table 3.

**Figure 9:**
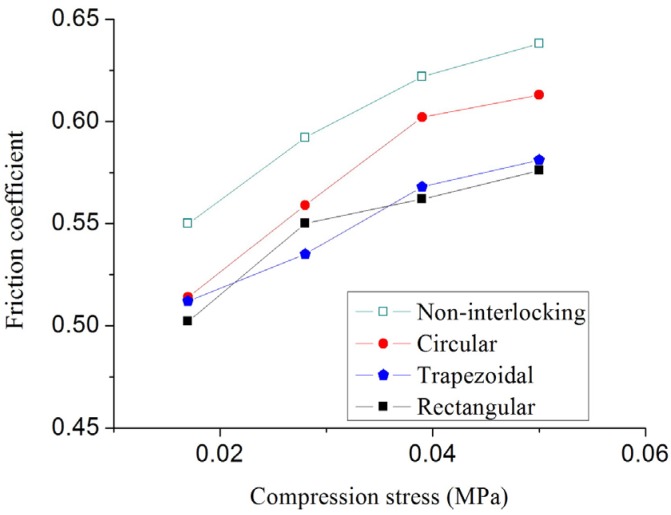
Effects of compression stress level on the friction coefficients.

According to Figure 9 and Table 3, compression stress level plays an important role in the friction coefficients of the N-IMB and IMB joints. Regardless of the interlocking shapes, as the compression stress increases, the friction coefficient increases by more than 12.5%, and the variation decreases. The maximum increment in the friction coefficient was observed for IMB with a circular shape (18%), and the minimum increment was achieved for IMB with a rectangular shape (12.5%). The friction coefficient is insensitive to the interlocking shape. Under the same compression stress, when the interlocking shape varies, the variation range of the friction coefficients is less than 10%.

**Figure 10:**
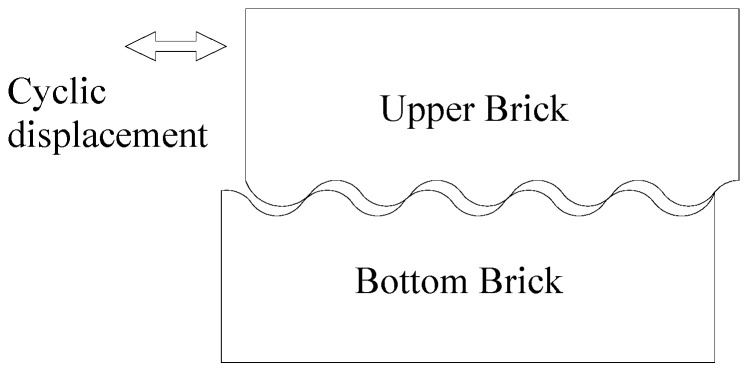
Sketch of the contact status for the MBs.

The effect of compression stress on the friction coefficients is primarily caused by the contact degree between two bricks. The upper and bottom bricks do not have a tight contact, because of the existence of “micro burrs”, as shown in Figure 10. The real contact area between the upper and bottom bricks varies as the compression changes. As the compression stress increases, more micro burrs bite into one another on the contact surface, resulting in an additional contact area and an increment in the shear force of the mortarless joints. Furthermore, with the increase in compression stress, the bite force is overcome during sliding, which also contributes to the increment in shear force.

### 4.3. Effect of Loading Cycles

The effects of loading cycles on the MB joints’ behavior were investigated by comparing the experimental results after four and 36 cycles. Both the friction coefficient and wear condition on the contact surface were investigated, and a constant compression stress level (0.05 MPa) was selected. The friction coefficients for N-IMB and IMB joints under different loading cycles are listed in Table 4.

The degradation rate (*DR*) of the friction coefficients was introduced to analyze the influence of the loading cycles quantitatively. *DR* can be calculated as:

(3) $$DR=\left|\frac{\mu_{36}-\mu_{4}}{\mu_{4}}\right|\times 100\%$$

where μ_4_ and μ_36_ are the friction coefficients obtained after four and 36 cycles, respectively. The *DR* for IMB with a circular interlocking shape has the minimum value (19%), which is close to the value of the N-IMB joints (21%). Meanwhile, the *DRs* for IMB joints with rectangular and trapezoidal interlocking shapes are much larger (with an average of 33%). The results indicate that *DR* increases with the decrease in the smoothness of the interlocking surface.

**Figure 11:**
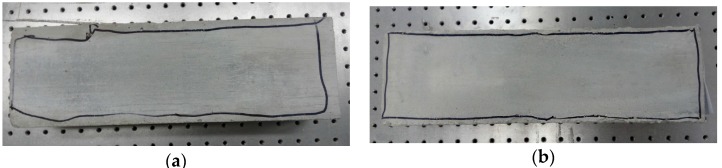

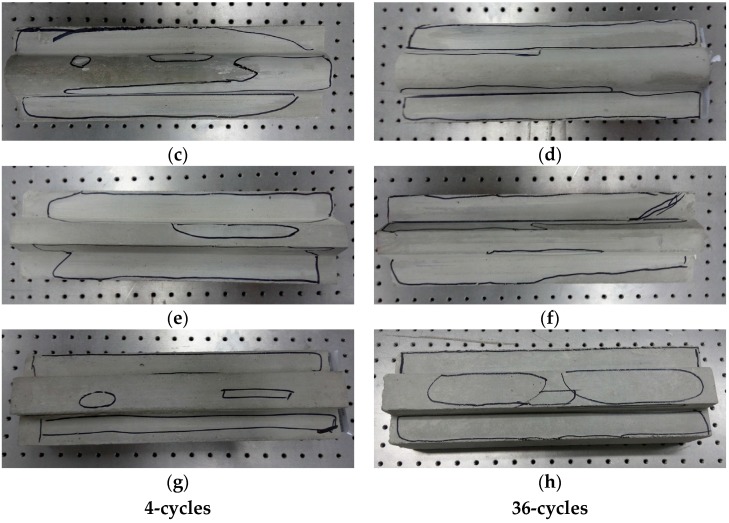
Photos of the wear condition for N-IMBs and IMBs after different loading cycles. (**a**) N-IMBs after four cycles; (**b**) N-IMBs after 36 cycles; (**c**) circular IMBs after four cycles; (**d**) circular IMBs after 36 cycles; (**e**) trapezoidal IMBs after four cycles; (**f**) trapezoidal IMBs after 36 cycles; (**g**) rectangular IMBs after four cycles; (**h**) rectangular IMBs after 36 cycles.

The wear conditions of the MB joints after four and 36 cycles are marked in Figure 11. For the specimens of N-IMB, the wear area was almost all around the contact surface (see Figure 11a) after performing four cycles of loading; only a slight increase in wear was observed after 36 cycles of loading, as shown in Figure 11b. For the specimens of IMB, as shown in Figure 11c–h, almost no wear was observed on the interlocking portion after four cycles of loading. However, as the loading cycles increased from four cycles to 36 cycles, more wear occurred. Compared to the research on DR, the newly-produced wear has a positive correlation with the DR of the friction coefficient, namely, when more wear occurs, a larger degradation ratio is obtained.

## 5. Conclusions

(1) A series of cyclic tests was carried out to investigate the compression-shear behavior of MB joints. In these tests, four different interlocking shapes, four compression stress levels and two loading cycles were investigated. A novel loading test methodology, which is convenient and accurate for cyclic tests, was applied.

(2) The hysteretic loops of the N-IMB and IMB joints under the shear-compression cyclic tests were obtained. A typical Mohr–Coulomb frictional behavior was observed in the N-IMB joints. A significant stiffness hardening effect was found for the IMB joints; the effect was mainly caused by the loading direction being not parallel to the longitudinal extension of the interlocking portion during loading.

(3) Compression stress level plays an important role in the friction coefficients of the N-IMB and IMB joints. Regardless of the interlocking shapes, as the compression stress increases, the friction coefficient increases by more than 12.5%, and the variation decreases. The friction coefficients are insensitive to the interlocking shapes. Under the same compression stress, when the interlocking shape varies, the variation range of the friction coefficients is less than 10%. The influence of compression stress on the friction coefficients is primarily caused by the contact degree between two bricks.

(4) The influence of loading cycles was examined by comparing the experimental results after four and 36 cycles. The wear condition was studied, and the degradation rate of the friction coefficient was defined. The results showed that as the loading cycle increases, the wear becomes increasingly severe. Wear condition has a positive correlation with the *DR* of the friction coefficient, which means that when more wear occurs, a larger *DR* is obtained.

(5) The circular IMB is the preferred choice for the MB panels. Firstly, the circular IMBs can be constructed easily in the engineering application; secondly, the circular IMB joints can absorb more in-plane energy dissipation and exhibit better out-of-plane behavior during the seismic action compared to the rectangular and trapezoidal IMB joints because of the maximum friction coefficient and the minimum *DR* of the friction coefficient due to the increase of loading cycles are obtained.

## Figures and Tables

**Table 1:** Mix proportion design of bricks. *w*/*c*: Water cement ratio.

Specimen	Cement (kg/m^3^)	Fly Ash (kg/m^3^)	Ceramsite (kg/m^3^)	Sand (kg/m^3^)	Water-Reducing Admixture	*w*/*c*	14-Day Compressive Strength (MPa)	Apparent Density (kg/m^3^)
1	300	50	430	780	1.2%	0.42	22.12	1843
2	300	50	500	650	1.2%	0.42	29.77	1792
3	280	70	560	600	1.2%	0.42	27.53	1700

**Table 2:** Density and compressive strength of bricks.

Specimen	Density (kg/m^3^)	28-Day Compressive Strength (MPa)
1-1	1779	33.2
1-2	1730	35.5
1-3	1799	32.5
2-1	1750	30
2-2	1756	30.8
2-3	1735	32.9
3-1	1754	32.3
3-2	1743	31.3
3-3	1728	30.5
4-1	1698	27.6
4-2	1706	32.8
4-3	1742	33.6
5-1	1777	31
5-2	1793	32.3
5-3	1759	31.1
6-1	1796	33.3
6-2	1699	30.4
6-3	1687	29
Average	1746	31.7

**Table 3:** Effect of compression stress on the friction coefficient.

Interlocking Shape	Compression Stress			
	0.017 MPa	0.028 MPa	0.039 MPa	0.05 MPa
Non-interlocking	0.55	0.592	0.622	0.638
Circular	0.514	0.559	0.602	0.613
Trapezoidal	0.512	0.535	0.568	0.581
Rectangular	0.502	0.55	0.562	0.576

**Table 4:** Effects of loading cycles on the friction coefficients.

Interlocking Shape	Loading Cycles		Degradation Rate (*DR*) (%)
	4 Cycles	36 Cycles	
Non-interlocking	0.638	0.502	21%
Circular	0.613	0.497	19%
Trapezoidal	0.581	0.405	30%
Rectangular	0.576	0.369	36%
